# 2800. Carbapenem Resistant *Enterobacteriaceae* bloodstream infections in children from Latin America & Caribbean region: systematic review and meta-analysis

**DOI:** 10.1093/ofid/ofad500.2411

**Published:** 2023-11-27

**Authors:** Silvina Ruvinsky, Carla Jimena, Macarena Roel, Martin Brizuela, Victoria Maria Portillo, Gabriela Naranjo-Zuniga, Rolando Ulloa-Gutierrez, Agustin Ciapponi, Daniel Comande, Ariel Bardach

**Affiliations:** Hospital de Pediatría Dr. Juan P. Garrahan, buenos aires, Ciudad Autonoma de Buenos Aires, Argentina; Hospital de Pediatria Juan P Garrahan, Capital Federal, Ciudad Autonoma de Buenos Aires, Argentina; Hospital de Pediatria JP Garrahan, Buenos Aires, Ciudad Autonoma de Buenos Aires, Argentina; Hospital Zonal General de Agudos "Dr Isidoro G Iriarte", Buenos Aires, Ciudad Autonoma de Buenos Aires, Argentina; Hospital de pediatría "Prof. Dr. Juan P. Garrahan", CABA, Ciudad Autonoma de Buenos Aires, Argentina; Hospital Nacional Niños, San Jose, San Jose, Costa Rica; Hospital Nacional de Niños Dr Carlos Saenz Herrera, San josé, San Jose, Costa Rica; IECS, Buenos Aires, Buenos Aires, Argentina; IECS, Buenos Aires, Buenos Aires, Argentina; IECS, Buenos Aires, Buenos Aires, Argentina

## Abstract

**Background:**

Systematic reviews about the burden and use of resource related to Carbapenem Resistant *Enterobacteriaceae* bloodstream infections (CRE-BI) in children from Latin America and Caribbean (LAC ) is scarce.

**Methods:**

**Objectives:**

To analyze demographic, epidemiological, clinical, use of resource and microbiological data from CRE-BI in LAC

**Methods:**

A systematic review following Cochrane methods, PRISMA/MOOSE statements was performed (PROSPERO 2022 CRD42022352504). We searched all databases (MEDLINE, Embase, LILACS, SciELO, CENTRAL, CINAHL, Cochrane Library, WHO Database and relevant websites) from 1/1/2012 to 9/30/2022. Independent selection, data extraction, and risk of bias assessment (RoB) were performed with COVIDENCE. Types of study included; observational/surveillance/experimental/quasi-experimental. Population: Inpatients < 19 years old, with CRE-BI. Outcomes: age, underlying conditions, previous (invasive procedures, colonization, antibiotic treatment, PICU). treatment and use of resources. Statistical analysis: proportion meta-analyses applying an arc-sine transformation and RoBTo, We conducted proportion meta-analyses and applied arc-sine. R studio was used

**Results:**

25/858 screened studies included for full text assessment with 243 patients were included. The most represented countries were Brazil (30%), Colombia (26%) and Argentina (22%). Median age was 34.5 months (IRQ 14-85). Predominant resistance mechanisms: KPC (96%), NDM (58%), OXA (27%). Pool proportion were: Male 55%. Underlying conditions 100% (immunocompromised host 84%), Previous Colonization with CRE was 42% (CI95% 13-78) and broad spectrum antibiotic used was 88% (CI95% 46-98%), most commonly carbapenems 84% (CI95% 72-91%) Bacteremia secondary to intra-abdominal infection was 80%. Combination antimicrobial treatment was 96% (CI95% 22-100%). Median length of stay 22.5 (IQR 22-30 days). Overall case fatality ratio was 48% (IC95% 27-69%).

**Conclusion:**

CRE-BI cause morbidity and mortality mainly in pediatric population with underlying disease in LAC region. Considering its high impact in public health, it's mandatory to establish adequate policies and programs to improve antimicrobial stewardship.

**Disclosures:**

**All Authors**: No reported disclosures

